# Seroprevalence of *Neospora caninum* in dairy cattle from north-west and centre of Romania

**DOI:** 10.1051/parasite/2011184349

**Published:** 2011-11-15

**Authors:** R.R. Gavrea, A. Iovu, B. Losson, V. Cozma

**Affiliations:** 1 University of Agricultural Sciences and Veterinary Medicine, Faculty of Veterinary Medicine, Parasitology Department Mănăştur Street, No. 3-5 400372 Cluj-Napoca Romania; 2 Université de Liège, Faculté de Médicine Vétérinaire, Parasitologie et Pathologie des Maladies Parasitaires Boulevard de Colonster 20 B-43a 4000 Liège Belgique

**Keywords:** neospororsis, cattle, dairy, Romania, néosporose, bovin, laiterie, Roumanie

## Abstract

Neosporosis is a disease that mainly affects cattle in both dairy and beef herds. The main definitive host of this parasite is the dog. Since 1984 and its first description a large number of data were published worldwide on this parasite. In Romania, the research regarding this parasite is limited. The purpose of this study was to determine the seroprevalence of *Neospora caninum* infection in dairy cattle from six regions in north-western Romania and to evaluate the intensity of infection in different animals groups. A total number of 901 samples (862 sera from adult cows and 39 sera from calves) were collected from dairy farms and were screened for the presence of specific IgG anti-bodies using an enzyme-linked immunosorbent assay (ELISA). The overall seroprevalence for neosporosis was 34.6%. In adult cows and calves seroprevalences reached 34.8% (300/862) and 30.8% for calves (12/39) respectively. In cattle which had previously aborted, seroprevalence was 40.9%. These results indicate that *N. caninum* infection is widespread among animals reared in dairy systems from Romania and a program for farmer training and a strategy for reducing the economic impact of the disease are needed.

## Abstract (fr)

La néosporose est une maladie qui affecte principalement les bovins laitiers et les élevages de bovins en général. Les hôtes définitifs de ce parasite sont représentés en majeure partie par les chiens. Depuis 1984, date à laquelle elle a été mentionnée en premier, un grand nombre de données ont été publiées à travers le monde. En Roumanie, les travaux de recherche sur ce parasite sont limités. Le but de cette étude était de déterminer la séroprévalence de l’infection par *Neospora caninum* chez des bovins laitiers de six régions du nord-ouest de la Roumanie et d’évaluer l’intensité de l’infection dans des groupes différents d’animaux. Au total, 901 échantillons (862 sérums provenant de vaches adultes et 39 sérums de veaux) ont été prélevés au hasard dans des fermes laitières et ont été examinés pour rechercher la présence d’anticorps IgG spécifiques de *N. caninum*, en utilisant un test immuno-enzymatique (ELISA). La séroprévalence globale pour la néosporose a été de 34,6 % : 34,8 % chez les vaches adultes (300/862) et 30,8 % chez les veaux (12/39). Chez les vaches ayant avorté auparavant, 40,9 % présentaient des anticorps contre *N. caninum*. Ces résultats montrent que l’infection à *N. caninum* est très répandue chez les animaux d’élevages laitiers en Roumanie, d’où la nécessité d’un programme de formation des agriculteurs et d’une stratégie afin d’en réduire l’impact économique.

Neosporosis is a disease caused by a protozoan parasite closely related to *Toxoplasma gondii*. As intermediate hosts, it affects mainly bovines causing abortion and birth of chronically infected calves that implies important economic losses mainly in dairy industry. The abortion can take place from the third month until the last month of pregnancy but mostly occurs at five-six months of gestation (Moore, 2005). The economical losses are determined by the pregnancy loss, lower production of milk, rebreeding of the animal and medical assistance and costs determined by the eventual replace of the infected animal (Dubey *et al.*, 2007). Other intermediate hosts can be represented by ovines, goats, dears, buffalos, and many others animals. As definitive hosts dogs, coyotes and Australian dingoes have been identified (McAllister *et al.*, 1998; Gondim *et al.*, 2004; King *et al.*, 2010). Worldwide the seroprevalence of neosporosis in cattle varies and different values were obtained: 5.7% in Japan (Koiwai *et al.*, 2006), 13.9% in Uruguay (Banales *et al.*, 2006), 11.3% in Venezuela (Lista-Alves *et al.*, 2006), 20.1% in Slovakia (Reiterova *et al.*, 2009), 24.1% in Spain (Panadero *et al.*, 2010) and 2.8% in Sweden (Loobuyck *et al.*, 2009).

In Romania the first report regarding N*eospora caninum* infection in cattle was in 2002 (Ionescu *et al.*, 2002) showing a prevalence of 20% (30/150 animals). The next year a larger study was in conducted and bovine sera were tested giving a seroprevalence of 33.71% (Ionescu *et al.*, 2003).

In this study a larger number of samples were taken under study and they were collected from a larger number of counties providing a much more reliable result regarding the spread of neosporosis in cattle from Romania.

## Materials and Methods

Between 2008 and 2010, a total number of 901 sera samples were collected from 16 dairy farms. These farms were located in six different counties from central and northwestern parts of Romania: Alba, Cluj, Maramureş, Mureş, Sibiu and Satu Mare. The lowest number of samples collected from a farm was six and the highest was 188. All the sampled farms had vaccination schemes against brucellosis, leptospirosis, infectious bovine rhinotracheitis and bovine viral diarrhea.

The blood was collected by puncture from jugular or caudal vein and, incubated at 37 °C and then centrifuged for serum collection. Sera were kept under - 20 °C until further use. All sera were tested for antibodies against *N. caninum* by using a commercial enzyme immuno-assay kit *Neospora caninum* Antibody Test Kit produced by IDEXX Laboratories, Switzerland. Briefly, each serum sample (diluted 1:100) was added to the *Neospora* antigen-coated microplates and incubated for 30 minutes at room temperature. Then, the antigen provided in the kit was added in each well and incubated at room temperature for another 30 minutes. The reaction was revealed by adding the chromogen and incubating for 15 minutes at room temperature. The color reaction was stopped by adding stop solution. Plates were read at 650 nm and the results were expressed as optical density (OD) values and were analyzed using the formulas provided by the manufacturer. Using a statistical package (EpiInfo Version 5) the p-value was also calculated.

## Results

Antibodies against *N. caninum* were detected in 312 (34.6%) serum samples from 901 pure breed cows. No results were considered as being doubtful. The average within-heard seroprevalence was 31.11%, with a range between 11.1 and 60.0% in seropositive farms (Table I). In adult cows and calves the seroprevalence were 34.8% (300/862) and 30.8% (12/39) respectively.

**Table I.** Seroprevalence of *N. caninum* in cattle form different counties of Romania.

Farm	Country	No. of samples collected	No. of positive samples	%	95% CI
1	Cluj	44	6	13.6	5.2 – 27.4
2		29	6	20.i	8.0 – 39.7
3		6	3	60.0	14.i – 94.i
4		96	28	29.2	20.3 – 39.3
5		34	13	38.2	22.2 – 56.4
6		67	13	19.4	10.8 – 30.9
i		7	3	42.9	9.9 – 81.6
8		35	11	31.4	16.9 – 49.3
9		8	1	12.5	0.3 – 52.7
10	Alba	35	9	25.i	12.5 – 43.3
11		144	49	34.0	26.3 – 42.4
12	Maramureç	9	1	11.1	0.3 – 48.2
13	Mureç	188	106	56.4	49.0 – 63.6
14		108	22	20.4	13.2 – 29.2
15	Sibiu	26	9	34.6	17.2 – 55.7
16	Satu Mare	65	31	4i.i	35.1 – 60.5

Total		901	312	31.11*	

* average within herd prevalence.

Out of the 901 animals tested, 137 (15,2%) had at least one abortion in their history. The seroprevalence of *N. caninum* infection in these animals reached a value of 40.9% (95% CI 32.6-49.6). Amongst the rest of the animals that had no previous reproduction problems the seroprevalence was 33.5% (256/764). The difference between these two animal categories was statistically significant: p < 0.05 (Table II).

**Table II.** Seroprevalence of *N. caninum* infection in cattle that had at least one abortion in their history.

Country	No. of samples collected	No. of animals that aborted / Prevalence of *N. caninum* infection in abortive cattle	No. of positive animals / Prevalence of neosporosis
Alba	179	36 / 30.6%	58 / 32.4%
Cluj	326	14 / 50.0%	85 / 26.1%
Maramureç	9	– / –	1 / 11.1%
Mureç	296	4 / 75.0%	128 / 43.2%
Sibiu	26	22 / 31.8%	9 / 34.6%
Satu Mare	65	61 / 45.9%	31 / 47.7%

Total	901	137 / 40.9%	312 / 34.6%*

* overall prevalence.

Also the tested animals were divided into two other categories: animals, which had no pregnancy, and animals that had at least one pregnancy in their history. From the first group, (176 animals), 77 presented antibodies against *N. caninum*, reaching a percentage of 43.8. From the second group (725 animals), 32.4% were positive. These results were also statistically significant: p < 0.004.

## Discussion

The seroprevalence of *N. caninum* infection in cattle was studied in many countries. It varied between countries and regions. In Romania, some other studies regarding neosporosis were performed and the prevalence obtained was of 56.2% (Gavrea *et al.*, 2008), 19.3% for cattle raised in extensive system (Gavrea & Cozma, 2009) and 55.95% in cattle with reproductive failure (Gavrea & Cozma, 2010).

The overall seroprevalence obtained in dairy cattle in six counties from Romania was 34.6% similar to that obtained in Thailand (34%) (Kyaw *et al.*, 2004), Costa Rica (43.3%) (Romero *et al.*, 2005) and Mexico (42%) (Garcia-Vazquez *et al.*, 2005). In the present herd seroprevalences varied between 11.1 and 60.0% and the differences were statistically significant between herds. The fact that all the farms taken under study had cattle positive for *N. caninum* infection indicates that this infection in dairy cattle is widespread in Romania. There have been many studies that analysed and demonstrated the association between seroprevalence of neosporosis and abortion. Although the association between abortion and *N. caninum* seropositivity was not strong (odds ratio, 0.72) there was some evidence of neosporosis-associated abortions. A relatively high seroprevalence of *N. caninum* was recorded in animals that aborted. There was also a significant difference between animals that had no recorded pregnancies and the ones that had at least one pregnancy in their history.
